# More than meets the I: the diverse antiviral and cellular functions of interferon-induced transmembrane proteins

**DOI:** 10.1186/s12977-017-0377-y

**Published:** 2017-11-21

**Authors:** Guoli Shi, Olivier Schwartz, Alex A. Compton

**Affiliations:** 1 Antiviral Immunity and Resistance Section, HIV Dynamics and Replication Program, Center for Cancer Research, National Cancer Institute, Frederick, MD USA; 2 Virus and Immunity Unit, Institut Pasteur, Paris, France; 3 UMR CNRS 3569, Paris, France

## Abstract

The first responders of human antiviral immunity are components of the intrinsic immune response that reside within each and every one of our cells. This cell-autonomous arsenal consists of nucleic acid sensors and antiviral effectors strategically placed by evolution to detect and restrict invading viruses. While some factors are present at baseline to allow for constant surveillance of the cell interior, others are upregulated by cytokines (such as interferons) that signal a viral infection underway in neighboring cells. In this review, we highlight the multiple roles played by the interferon-induced transmembrane (IFITM) proteins during viral infection, with focuses on IFITM3 and HIV-1. Moreover, we discuss the cellular pathways in which IFITM proteins are intertwined and the various functions they have been ascribed outside the context of infection. While appreciated as broadly-acting, potent restriction factors that prevent virus infection and pathogenesis in cell culture and in vivo, questions remain regarding their precise mode of action and importance in certain viral contexts. Continued efforts to study IFITM protein function will further cement their status as critical host determinants of virus susceptibility and prioritize them in the development of new antiviral therapies.

## The earliest-acting restriction factors against multiple pathogenic viruses

The IFITM protein family is encoded by five genes in humans, including the immune-related *IFITM1*, *IFITM2*, and *IFITM3*, as well as *IFITM5* and *IFITM10* which have no characterized roles in immunity [1]. Today, *IFITM* genes are present in many vertebrate animal species yet they likely emerged in early unicellular eukaryotes via horizontal gene transmission from a bacterium [2]. Since then, species-specific gene expansions have given rise to unique *IFITM* gene repertoires that vary at the level of sequence and copy number [3–5]. In addition to the canonical *IFITM* gene locus on chromosome 11 in humans, there are a number of *IFITM*-like genes dispersed throughout our genome for which a functional understanding is lacking [2]. Despite what their name implies, only the immune-related *IFITM* genes are interferon-inducible, and furthermore, moderate to high levels of expression may be seen in several tissue types even in the absence of interferon.

The immunological and clinical importance of IFITM proteins to innate immunity is tied to their unique ability to inhibit the earliest step of the virus life cycle: entry into cells. As a result, they prevent not only viral replication but also the sequelae of virus-associated disease, such as cytopathicity (cell death) and inflammation. Initially revealed to be endogenous inhibitors of Influenza A virus (IAV), West Nile virus (WNV), and Dengue virus, today it is recognized that a growing list of viruses are sensitive to IFITM-mediated restriction [6, 7]. The use of retroviral “pseudotypes,” in which different viral envelope glycoproteins are swapped into the same retrovirus capsid core, demonstrated that the route of cellular entry is a major determinant of restriction and identified strains exhibiting resistance. In general, those that require a pH-dependent triggering of viral fusion machinery in endosomes are the most affected when IFITM proteins, in particular IFITM3, are overexpressed or silenced in cells [8–10]. These findings suggested that IFITM-mediated antiviral activity manifests at the entry stage.

The subcellular localization of IFITM proteins is important to our understanding of how virus entry is inhibited. They are regularly detected in endosomes, lysosomes, autophagosomes, and the plasma membrane, and the extent of which can vary by cell type and by IFITM family member. Their presence at various cellular compartments is the result of dynamic protein trafficking that begins with de novo synthesis in the endoplasmic reticulum and ends with degradation in lysosomes [11]. Multiple pieces of evidence suggest that IFITM3 takes on a type II transmembrane protein topology, with a cytosolic amino-terminus, a luminal/extracellular carboxy-terminus, and two hydrophobic domains: one intramembrane domain (HD1) and one transmembrane domain (HD2) [12–14]. Interestingly, there is also support for alternative membrane topologies in which the termini orientations are reversed [13]. The hydrophobic domains of IFITM members ensure movement between membrane-enclosed vesicles in the biosynthetic-secretory pathway, and post-translational lipidification with an S-palmitoyl group promotes durable membrane associations [15, 16]. After passage through the endoplasmic reticulum, golgi complex, and plasma membrane, endocytic sorting motifs in the amino-terminus of IFITM2 and IFITM3 allow internalization into endosomes, and this positioning is crucial for antiviral activity [5, 17, 18]. Late endosomes, multivesicular bodies, and lysosomes form hybrid organelles in cells and are nearly indistinguishable by conventional methods, and hence the subcellular localization of IFITM3 is often described as endolysosomal. However, some of the overlap with lysosomes must be attributed to the degradative pathway controlling IFITM3 protein turnover, which is mediated by the E3 ubiquitin ligase NEDD4 [19]. Furthermore, recent reports highlighting roles for IFITM3 in autophagy, a catabolic process involving the degradation of cellular cargo in lysosomes, may explain the apparent association with autophagosomes [20]. In summary, while endolysosomes appear to be the key compartment whereby IFITM3 restricts virus entry, its detection in other organelles may be indicative of uncharacterized cellular functions that are only now being investigated.

## Preventing virus entry at the level of cytoplasmic access

**Fig. 1:**
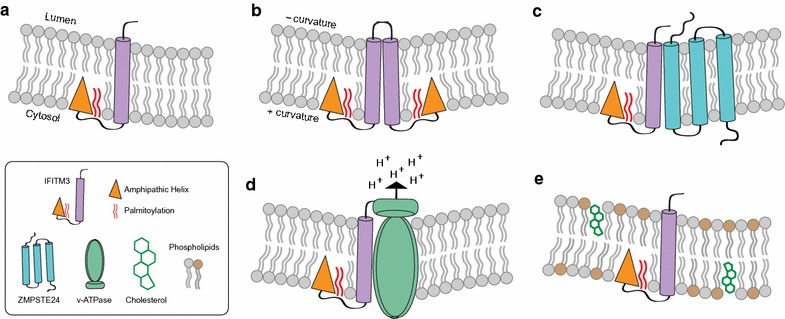
The various mechanisms by which IFITM proteins may inhibit virus-cell fusion. **a** A simplified membrane topology model of IFITM3 is represented, with emphasis made on the amphipathic helix of hydrophobic domain 1, neighboring palmitoylated cysteine residues, and the transmembrane helix of hydrophobic domain 2. In this illustration, the amino-terminus faces the cytosol while the carboxy-terminus faces the ER/endosomal lumen or extracellular space, but other conformations may exist. **b** IFITM3 multimerization is important for antiviral activity and cell-based experiments indicate that IFITM3 augments membrane rigidity and instills positive curvature, as defined from the vantage of the cytosol. **c** IFITM3 may indirectly inhibit virus entry via an association with other membrane proteins, such as ZMPSTE24. Only three of seven transmembrane domains of ZMPSTE24 are indicated. **d** An effect of IFITM3 on the trafficking and/or function of the vacuolar ATPase (v-ATPase) has been reported, raising the possibility that IFITM3 indirectly inhibits virus entry by increasing endosomal acidity. **e** IFITM3 may influence the cholesterol content of endosomes, which has been shown to affect virus-cell fusion events. The actions of IFITM3 on virus entry and on cholesterol levels have been dissociated in several studies, but further impacts on membrane lipid content still await testing

As residents of cellular membranes, an attractive explanation for the effects of IFITM proteins on virus entry involves direct modification of membrane rigidity and curvature, but other indirect mechanisms have also been proposed (Fig. 1). It is well recognized that integral membrane proteins can alter the shape and fluidity of lipid bilayers, and furthermore, that virus-cell fusion is affected by these factors [21]. Most of what we know about the antiviral activities of IFITM proteins results from work using IAV and vesicular stomatitis virus (VSV), which perform pH-dependent fusion reactions in endosomes to gain access to the cell interior [22]. Early findings using fluorescent microscopy and flow cytometry of single cells implicated IFITM3 as an obstacle to virus-cell fusion. When cells overexpressing IFITM3 are challenged with IAV, productive infection is inhibited [8]. Upon close examination, virions undergo attachment and internalization into cells before becoming cleared from the cell interior [9]. Parallel experiments further showed that IFITM3 may expand the size and acidity of the endolysomal compartment itself. In effect, IFITM3 appears to trap endocytosed virions inside vesicles slated for destruction in a degradative pathway [6]. It remains to be determined whether IFITM3-containing structures represent a distinct, hostile subset of endolysosomes that are not conducive to virus-cell fusion. Indeed, an article reported that IAV sensitivity to IFITM3 is determined by the acidic threshold at which viral hemagglutinin (HA) triggers virus-cell fusion in endosomes. That is, HA variants which drive fusion at higher pH (less acidic) are more resistant to the block by IFITM3, suggesting that evasion of more acidic, late endosomes (where IFITM3 resides in most cell types) enables infection [23]. This assertion is supported further by the observation that VSV infection, which requires virus-cell fusion in early endosomes, is less affected by the presence of IFITM3 [8, 22, 24]. The effects of IFITM proteins on the makeup of the cell interior may present important clues about its principal mechanism of action [9].

Simple experiments examining cell–cell fusion, in which IFITM proteins and viral envelope proteins are co-expressed, have been instrumental in testing different mechanistic possibilities. Initial reports showed that IFITM members inhibit cell–cell fusion mediated by three classes of viral fusion proteins (some more than others) in a way that did not affect envelope expression itself [25]. This finding suggests that IFITM proteins may affect the physical aspects of membranes in a way that prevents fusion by diverse viral fusion proteins. While informative, an important caveat is that cell–cell fusion experiments in tissue culture do not accurately reproduce the true nature of virus-cell encounters, since IFITM and viral fusion proteins may not be expressed at physiologically relevant levels or at correct subcellular sites. Attempts at identifying the precise step of the fusion sequence inhibited by IFITM suggested a block prior to hemifusion, the point at which lipid mixing begins between leaflets of two juxtaposed membranes [25]. However, other techniques including virus particle tracking detected lipid mixing between cell and virus in the presence of IFITM but no viral escape into the cytoplasm, suggesting that dilation of the fusion pore is restricted [26]. The basis for these observations may lie with increased lipid density or increased positive curvature of IFITM-containing membranes [25]. Another piece of evidence in support of IFITM proteins as membrane remodelers is the finding that amphotericin B, an antifungal compound known to enhance membrane fluidity, counteracts the activity of IFITM3 to render cells permissive to infection [27]. Furthermore, the discovery of an amphipathic helix within the first hydrophobic domain of IFITM3 was found to be crucial to the inhibition of virus entry [14]. This previously unappreciated structure is positioned adjacent to palmitoylated cysteine residues involved in membrane targeting and is also important for the inhibition of cell–cell fusion. Based on previously recognized functions of other proteins endowed with an amphipathic helix, IFITM proteins may sense membrane changes occurring during virus-driven hemifusion and prevent fusion pore dilation. These latest findings implicate the first hydrophobic domain (HD1) of IFITM3 as the functional “arm” responsible for its antiviral activity. However, this contrasts with a previous finding that proposed a role for HD2 in cholesterol augmentation in endosomes [28]. Residues within HD2 of IFITM3 were reported to interact with the vesicle-associated membrane protein-associated A (VAPA), previously linked to cholesterol trafficking. While the effects of cholesterol on membrane fluidity and virus-cell fusion are well-characterized, a number of studies have failed to draw a mechanistic link between IFITM3 activity and cholesterol levels [26, 27, 29, 30]. Therefore, effects of IFITM3 on lipid trafficking may not underlie virus inhibition, yet may be indicative of presently uncharacterized activities in the cell. Interestingly, VAPA and the related protein VAPB can enhance the replication of some viruses, most likely through the manipulation of the lipid composition of cell membranes used for virus replication [31, 32].

Overall, the study of pathogenic RNA viruses demonstrating a high degree of sensitivity to IFITM-mediated restriction has provided some mechanistic insight behind the block they impose to virus entry. Nevertheless, the demonstration that certain viruses are resistant to, or even benefit from, the IFITM proteins indicates that antiviral activity may be achieved through the coordination of other cellular proteins. IFITM proteins, especially IFITM1, are known to reside in the plasma membrane and to interact with transmembrane proteins, such as tetraspanins. Hepatitis C Virus (HCV) utilizes CD81 and occludin as co-receptors, which are components of cellular tight junctions and also happen to interact with IFITM1 [33]. Overexpression of IFITM1 leads to inhibition of HCV entry into cells as well as disruption of tight junction complexes, suggesting that these two effects are functionally linked [34]. Since CD81 and other tetraspanin proteins can modulate fusion events in multiple virus infections [35–38], the ability for IFITM proteins to alter the location or clustering of membrane protein complexes may be central to their antiviral activity. An ‘indirect’ mode of action is in agreement with the observation that cellular entry of the coronavirus HCoV-OC43 is promoted by IFITM proteins [39]. This exceptional case may be explained by the fact that different viruses take advantage of distinct cellular receptors and thus distinct entry routes that are differentially impacted by the presence of IFITM proteins.

A transmembrane metalloprotease known as ZMPSTE24 (also known as FACE1) has recently emerged as a downstream effector of IFITM3 [40]. The authors posit that IFITM3 traffics ZMPSTE24 to the sites of virus fusion at endosomes via a direct protein–protein interaction. Importantly, in ZMPSTE24 knockout cells, IFITM3 overexpression no longer provides protection from virus challenge. No mechanistic information is available for the antiviral activities of ZMPSTE24 and thus future studies must address the specific determinants of how it binds to IFITM3. It is possible that domains of IFITM3 previously shown to be essential for antiviral function play roles in modulating the location or function of this and other cellular factors, resulting in an organized block to virus entry.

## Early-stage lentivirus inhibition: IFITM in target cells

The path to identifying the roles played by IFITM proteins during HIV-1 infection was less straightforward for multiple reasons. First, while the impact of IFITM proteins on viruses that require endocytosis and pH-dependent fusion is clearly appreciated, the role for endocytosis in HIV-1 cellular entry has been widely debated [41]. Second, HIV-1 exhibits the capacity to spread between cells in a process known as cell-to-cell transmission, which likely allows escape from immune barriers [42]. Despite these challenges, the study of IFITM and HIV-1 was mutually instructive: a new antiviral function was described for the former and the ports of cellular entry were defined for the latter.

In the first formal description of the antiviral properties of IFITM family members, IFITM3 silencing had little to no effect on HIV-1 infection in HeLa-CD4 cells, thus grouping it with another retrovirus (amphotropic Murine Leukemia Virus) deemed to be resistant [8]. Also, in the paper that identified tetherin/BST-2 as the target of viral accessory protein Vpu, which promotes HIV-1 release from cells, it was shown that IFITM proteins exhibited no impact on HIV-1 egress [43]. However, two high-throughput screens studying the activities of interferon stimulated genes (ISGs) provided evidence that IFITM proteins impact HIV-1 replication when silenced or overexpressed [44, 45]. Lu et al. provided the first in-depth functional demonstration of IFITM-mediated HIV-1 restriction at the level of entry in T cells, and this work was later extended to other lentiviruses of non-human primates [46]. In addition, experiments allowing ongoing replication in tissue culture revealed that the antiviral properties of IFITM proteins may not be limited to the inhibition of virus entry (see next section).

As the primary determinant for virus-cell attachment and the subsequent fusion reaction, the viral envelope glycoprotein (Env) was suspected to play an important role in whether or not HIV-1 and related lentiviruses are subject to inhibition by IFITM proteins. A report taking advantage of retroviral Env pseudotyping provided key insight into viral factors that govern susceptibility and resistance. The authors showed that one can decrease sensitivity to IFITM proteins by increasing amounts of lentiviral Env incorporated into virions [47]. However, they pointed out that the capsid core (vector) is also a determinant, indicating that the structure of the virus-like particle and/or its coordination with Env also affects the degree of restriction. Another study corroborated the importance of Env with regards to inhibition by IFITM proteins, this time via examination of patient-derived HIV-1 clones known as transmitted/founder (TF) strains [48]. These viral variants represent a close approximation of the viral sequence that seeds infection in a newly-infected individual, and they tend to utilize a specific co-receptor on the cell surface known as CCR5. Here, it was revealed that the sensitivity of HIV-1 entry to IFITM-mediated restriction depends on coreceptor usage and the subcellular localization of IFITM in the host cell. CXCR4-tropic HIV-1 strains were shown to exhibit sensitivity to IFITM2 and IFITM3, which are mostly localized to endolysosomes, while CCR5-tropic strains were sensitive to IFITM1 at the plasma membrane. This differential outcome suggests that HIV-1 fusion may occur in endosomes or at the plasma membrane depending on which virus coreceptor is engaged at the cell surface. TF strains are relatively resistant to IFITM-mediated restriction, yet matched viral clones derived 6 months following initial infection exhibited a gain of sensitivity to IFITM2 and IFITM3 [49]. This finding suggests that founder viruses enter cells at the plasma membrane, while viruses isolated at later stages of infection might increasingly rely on endosomal entry. However, IFITM2 and IFITM3 also transit to the plasma membrane before endocytosis, and thus the varying sensitivity of HIV-1 strains may result from fusion at different plasma membrane microdomains. Furthermore, the authors show that the site of HIV-1 entry, as inferred by sensitivity to IFITM proteins, may also depend on cell surface levels of CD4. Another report reinforced the link between IFITM and HIV-1 entry by demonstrating that CXCR4-tropic, but not CCR5-tropic HIV-1, is hypersensitive to a splice variant of IFITM2 lacking the amino terminus. Notably, this IFITM variant is especially abundant in primary human cells (CD4+ T cells and monocytes) that serve as targets for HIV-1 infection in vivo [50]. Together, these recent data suggest that IFITM2 and IFITM3 may be major selective pressures responsible for the use of CCR5 during primary HIV-1 infection.

## Late-stage lentivirus inhibition: IFITM in virus-producing cells

In addition to restricting virus entry, recent findings indicate that IFITM proteins perform antiviral functions impacting late stages of the HIV-1 life cycle. In contrast to previous strategies in which infections were launched using only cell-free virus preparations, the use of cell co-culture experiments using infected cells (donors) and uninfected cells (targets) revealed new antiviral functions [51]. We found that IFITM overexpression in targets had little to no consequence for HIV-1 transmission and spread in this context, while, surprisingly, overexpression in donor cells led to potent decreases [51]. Further experiments showed that the block to virus spread is attributed to an inhibition of virion infectivity, with IFITM3 exhibiting the most potent restriction. Virus-cell fusions assays showed that HIV-1 virions produced in the presence of IFITM3 are less fusogenic when incubated with fresh target cells, and assessment of virion content indicated that IFITM3 incorporates into the viral lipid bilayer. This antiviral activity is enhanced upon expression of an IFITM3 mutant that is defective for endocytosis, indicating that restriction of HIV-1 virion infectivity is performed at the plasma membrane [5]. Two other teams reported similar findings on this phenomenon in HIV-1 producing cells, with one adding a layer of mechanistic insight involving HIV-1 Env glycoprotein [52–54]. Here, the production of HIV-1 particles in the presence of IFITM3 via co-transfection of 293T cells resulted in defects in Env maturation and decreases in virion-associated gp120, the infectious form of Env [53]. The authors posited that IFITM3 interferes with Env via a protein–protein interaction in virus-producing cells. Of note, this relationship between antiviral protein and Env appears to hold true upon examination of non-human primate IFITM3 and their lentiviral counterparts [54]. Nonetheless, there exist certain discrepancies that cloud the potential importance of this observation. First, IFITM members inhibit HIV-1 infectivity to varying degrees (IFITM3 > IFITM2 > IFITM1) [51] yet all three proteins inhibit certain Env proteins to a similar extent [55]. Second, IFITM3 overexpression in T cells reduces virus infectivity but has no detectable impact on HIV-1 Env levels in infected cells or purified virions [51], and a lack of effect has been reported by others using diverse experimental systems [5, 48, 56]. Third, we now know that the negative “imprinting” of virions by IFITM3 occurs with various DNA and RNA viruses, apparently in the absence of envelope glycoprotein perturbation [56]. Therefore, follow-up experiments will require the study of endogenous IFITM3 in various cell types as well as the effects of type-I interferon on HIV-1 Env maturation and virion incorporation. Overall, it is unclear whether modification of Env is directly responsible for the inhibition of HIV-1 virion infectivity, nor is it known whether the virion incorporation of IFITM3 is critical for this effect. It is possible that both play mechanistic roles which are not mutually exclusive. Of note, enriched plasma membrane localization of IFITM3 enhances both anti-HIV-1 activity as well as IFITM3 incorporation into virions, suggesting a functional link [5]. Nonetheless, the recent identification of Env variants that are resistant to the IFITM3-mediated restriction of virion infectivity confirms this viral protein as an important determinant. It was shown that a particular CCR5-tropic strain (AD8) of Env is unaffected by IFITM3 overexpression in virus-producing cells, exhibiting no defects in virion infectivity. This resistance phenotype maps to the V3 loop of gp120 and is transferrable to other Env isolates [57]. Another feature of HIV-1 strains that are most sensitive to the IFITM3-imposed block to virus fusogenicity is the use of Env proteins with high sensitivity to both soluble CD4 and the neutralizing antibody 17b, which recognizes a CD4-induced epitope. Therefore, virus susceptibility to the infectivity defect caused by IFITM3 is linked to conformational changes in the CD4-binding site of Env, although the stability or clustering of virion-incorporated Env trimers may also be involved. Based on what we know about the changes to cellular membrane fluidity caused by IFITM proteins, it is possible that similar disturbances in the viral lipid bilayer containing Env are involved in the restriction of virion infectivity.

Another important observation made in these studies is that resistance to one antiviral function of IFITM3 is associated with resistance to another, since the AD8 strain exhibits relatively less sensitivity to the effects of IFITM3 in both target cells and producer cells, and TF strains are completely resistant to both modes of restriction [48, 56]. Thus, IFITM3 performs at least two antiviral activities against HIV-1 for which there may be mechanistic overlap. For reasons that are not yet clear, CD4 engagement by Env is an important determinant for whether virus is restricted at the level of target cells and producer cells [48, 55]. It is possible that CD4 binding and the conformational changes in Env that follow (which dictate interactions with co-receptors) affect the route and kinetics of HIV-1 entry into cells [58], and in turn, affect sensitivity to IFITM-mediated antiviral activities. Globally, the multiple constraints that IFITM proteins place on HIV-1 Env suggest that these cellular factors contribute to the genetic bottleneck that selects for interferon-resistant virus at the earliest stages of HIV-1 infection in vivo [59].

## The extended immunological impact of IFITM: beyond the endosome

The study of diverse viruses has been central to discoveries involving the IFITM protein family. The physiological importance of IFITM activity has been clearly demonstrated for those viruses that exhibit the greatest sensitivity in cell culture experiments, such as IAV. However, additional functions were found using viruses that are actually resistant to the block at cellular entry. In both cases, the use of transgenic mice deficient for the murine *ifitm* locus was central to establishing the in vivo significance of IFITM proteins in diseased and healthy states.

Downregulation of IFITM3 in target cells can lead to increased cytopathicity upon virus challenge in vitro, which has been attributed to its role as a protective barrier preventing virus entry and subsequent replication [60, 61]. Nonetheless, in vivo experiments using mouse-adapted virus strains have been critically important to understanding the full spectrum of downstream consequences resulting from *ifitm* deletion, ranging from cell death to the manifestation of virus-associated disease propagated throughout the host organism. For example, accelerated disease progression and mortality are observed in IFITM3-deficient mice challenged with IAV, suggesting that IFITM3 confers a survival advantage to cells exposed to virus [62, 63]. Indeed, the expression of IFITM3 may preserve the integrity and function of cells that would otherwise be destroyed by uninterrupted viral replication [64]. It was reported that elevated levels of IFITM3 protein in memory CD8+ T lymphocytes, which kill virus-infected cells and serve as important targets themselves for IAV infection in the lung, promotes their survival during infection and enables long-term defense against future viral exposures [65]. In a murine model of Chikungunya virus (CHIKV), mice lacking IFITM3 sustained greater joint swelling which was correlated to increased viral burden and pro-inflammatory cytokine production [66]. IFITM3-deficient mice were also found to be more vulnerable to lethality in the context of WNV. While WNV disease is associated with neurotropism, IFITM3 limited pathogenesis by suppressing viremia first in peripheral organs [67]. Thus, by acting as the first line of defense in cells exposed to invading viruses, IFITM proteins prevent a plethora of adverse events that can lead to disease and death.

Yet unexpected antiviral activities associated with IFITM3 were revealed during murine cytomegalovirus (CMV) infection. In this case, IFITM3 does not limit virus entry into cells (the protein was previously shown to facilitate CMV virion morphogenesis [68]). Rather, CMV infection in IFITM3-deficient mice led to much higher production of the pro-inflammatory cytokine interleukin-6 (IL-6) [69]. The result was dysregulation of cellular immunity and impaired control of virus replication, suggesting that murine IFITM3 plays a part in regulating cytokine production important for resolution of virus infection. Another example in which IFITM3 could be found performing a non-canonical activity was in the setting of Sendai Virus (SeV) infection. This murine paramyxovirus has been shown to be insensitive to the IFITM3-mediated block to virus entry [70]. Notwithstanding, it was shown that IFITM3 regulates interferon-beta production triggered by SeV infection in human cell lines [71]. That is, overexpression of IFITM3 inhibited production of the cytokine while knockdown had the opposite effect. The authors propose that IFITM3 associates with the transcription factor driving interferon-beta gene expression, IRF3, and accelerates its turnover in autophagosomes. Since interferon-beta itself induces the expression of IFITM3, this implies a role in negative feedback of the interferon pathway [71]. This finding warrants testing in an in vivo murine model, but it seems likely that the effects of IFITM proteins on interferon signaling will yield much interest from researchers and clinicians in the field of microbial pathogenesis.

Naturally occurring variation in *IFITM* genes has provided an additional genetic platform for the study of pathogenic virus infections important to human public health. Population-level associations have been drawn between single-nucleotide polymorphisms (SNP) in *IFITM3* and severe outcomes following IAV infection, corroborating an important in vivo function but lacking a mechanistic explanation. The most cited example is rs12252-C, a nonsynonymous mutation in the first coding exon of IFITM3, which was predicted to affect mRNA splicing and to produce protein with an amino-terminal truncation [63]. This minor variant was found to be enriched in a group of individuals hospitalized following IAV infection, and it is found at relatively high frequency among Asian ethnic groups. However, the reasons for the disease association has remained elusive, as the truncated isoform of IFITM3 predicted to result from the SNP had not been detected in cells or individuals. It has now been reported that cell lines derived from individuals who are homozygous for rs12252-C express a mRNA transcript encoding full-length IFITM3, whereas the shorter isoform was not found [50]. Over the course of several years, a slew of publications has either confirmed or refuted the genetic association between rs12252-C and IAV infection outcome.

A recent and comprehensive study now links an additional variant in the *IFITM3* locus to severe influenza-associated illness in three independent cohorts, albeit the SNP identified is distinct from rs12252-C. Known as rs34481144-A, it is found in the 5’ untranslated region and is linked to lower IFITM3 protein levels in cells [72]. Experimental evidence showed that the SNP controls gene promoter activity via decreased binding of transcription factor IRF3 and increased binding of CTCF, which promote and repress *IFITM3* transcription, respectively. In agreement with the previous finding that IFITM3 preserves antiviral CD8 + T cells [65], individuals harboring rs34481144-A contained reduced numbers of these cells in lung airways during infection [72]. The evolutionary pressures responsible for the maintenance of this ‘defective’ allele in humans are unclear, but its existence may allude to the involvement of IFITM3 in cellular processes requiring fine-tuned protein expression.

## Moonlighting in the membranes

Before it was realized that IFITM proteins perform broad-spectrum antiviral activities, they were implicated in pathways important to embryonic development and cancer (exhaustively reviewed in [73]). A crucial contribution to developmental processes seems unlikely, since transgenic mice in which the murine *ifitm* locus is knocked out exhibit no obvious abnormalities apart from being fat, suggesting a metabolic irregularity [74]. There is ample evidence, on the other hand, for both positive and negative regulation of *IFITM* expression during tumorigenesis. While detailed descriptions were previously lacking, recent developments have provided a mechanistic grounding to many observations linking IFITM proteins to cell proliferation, adhesion, and migration.

**Fig. 2:**
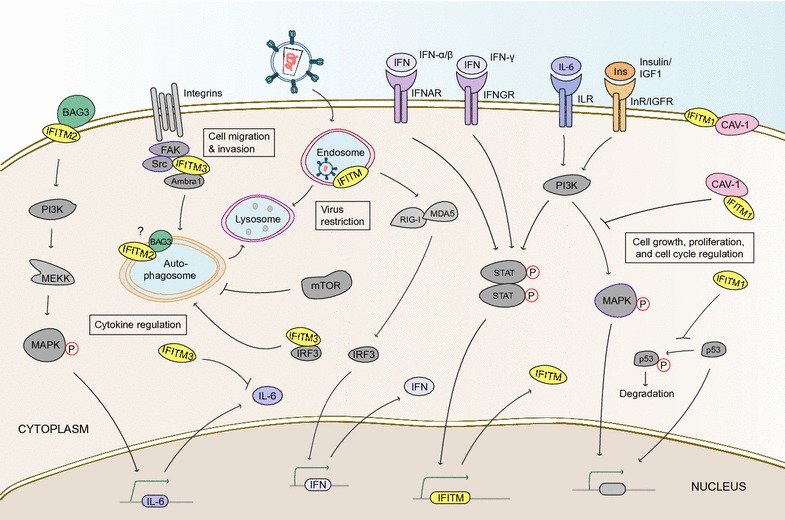
IFITM proteins are involved in various cellular processes with direct and indirect impacts on immunity. *Virus restriction*; In the event of successful virus invasion, nucleic acid sensors including RIG-I and MDA5 are triggered and IRF transcription factors are activated to induce the production of type-I interferons, which, in turn, initiate transcription of *IFITM* genes and other interferon-stimulated genes. IFITM proteins restrict the cytosolic access of virions undergoing endocytosis, possibly via inhibition of virus-cell fusion and destruction in an endolysosomal pathway. *Cytokine regulation*; IFITM3 is a negative regulator of the interferon response because it accelerates the turnover of IRF3 in autophagosomes. It also suppresses the production of IL-6, a pro-inflammatory cytokine which, itself, can also induce the expression of *IFITM* genes. IFITM2, in contrast, promotes the upregulation of IL-6 by acting as a cell surface receptor for secreted BAG3. As BAG3 is also a well-characterized chaperone for selective autophagy, it will be of interest to determine if IFITM2 also participates with BAG3 in autophagy-related processes. *Cell migration and invasion*; IFITM3 is a central component of a multi-protein interaction involving Src, FAK, and Ambra1, which is important for regulating cell adhesion and movement. IFITM3 assists in the subcellular trafficking of Src between focal adhesion points and autophagosomes. *Cell growth, proliferation, and cell cycle regulation*; Interferon signaling is known to negatively regulate cell division and growth via STAT signaling, with IFITM proteins serving as downstream effectors. IFITM1 interacts with caveolin-1 (CAV-1) to inhibit ERK/MAPK signaling, a pathway which stimulates cell proliferation when active. IFITM1 also stabilizes p53, a tumor suppressor with anti-proliferative functions

In addition to the interferon signaling pathways, *IFITM* gene expression is controlled by a number of cascades involved in cellular homeostasis (Fig. 2). For example, growth factor receptors lead to downstream upregulation of IFITM2 upon triggering by insulin-like growth factor-1 (IGF1), which relies on signaling via phosphatidylinositol-3-kinase (PI3K) and Akt kinase. In gastric cancer cohorts, the upregulation of IFITM2 is associated with accelerated disease progression and shorter survival time [75]. Silencing of IFITM2 in gastric cancer cells decreased cell proliferation, migration, and metastasis, while IFITM2 depletion in a mouse model resulted in dramatic decreases in tumor size [75]. IFITM3 has also been functionally implicated in this cancer type, as its knockdown suppressed tumor cell migration, invasion and proliferation capacity [76]. There is also evidence that IFITM3 acts indirectly to affect these cellular properties through regulation of other cellular proteins, such as osteopontin [77]. Recently, the involvement of IFITM3 in cancer was further extended to include spatial regulation of the Src oncoprotein. The trafficking of Src between focal adhesions and the cell interior, which is regulated by activating molecule in Beclin1-regulated autophagy (Ambra1) and focal adhesion kinase (FAK), is important for cell migration and metastasis. Ambra1 redirects Src towards autophagosomes to disfavor substrate attachment and favor cell movement in an IFITM3-dependent manner [78] (Fig. 2). Collectively, the twin antiviral proteins IFITM2 and IFITM3 may serve as biomarkers for tumorigenic phenotypes as well as targets for anti-cancer interventions. It remains to be determined which domains of IFITM proteins are involved in the control of cellular properties and if they overlap with those known to be involved in antiviral immunity. For example, just as the subcellular localization of IFITM proteins is important to their antiviral activities [11], it may also affect their involvement in cellular housekeeping functions. As a proof of principle, IFITM2 can act as a cell surface receptor for a secreted form of BAG3, which promotes pro-survival signaling through the  PI3K and p38 MAPK pathways [79].

Several reports highlighting IFITM-mediated impacts on basic cellular processes provide further opportunities to test how these proteins inhibit virus infections. A focus on functional roles of endogenous, rather than overexpressed, IFITM proteins has been crucial to this end. A prime example is the observation that deletion of the *ifitm* locus in murine cells led to interruption of clathrin-mediated endocytosis and loss of acidity within endosomes, suggesting that endogenous IFITM proteins might positively regulate these processes in vivo [80]. This possibility is further supported by work in astrocytes showing that knockdown of IFITM3 inhibits clathrin-dependent endocytosis [81]. As previously mentioned, the *ifitm*-deficient mice exhibit an age-related obesity associated with defects in leptin signaling, which could be explained by disturbances in ligand-receptor internalization [74]. Interestingly, the IFITM proteins are also involved with the endocytosis-associated protein caveolin-1 (CAV-1), with consequences for cell signaling events [82, 83]. Together, these data hint that impacts on endocytic trafficking may also contribute to the mechanisms by which IFITM proteins inhibit virus entry. While the antiviral effects of IFITM proteins are generally assumed to manifest at the stage of virus-cell fusion, these data suggest that an effect on virus internalization must be carefully considered on a case-by-case basis. For example, since knockdown or knockout of *IFITM3* leads to decreases in clathrin-mediated endocytosis and increases in virus entry, it is possible that IFITM3 promotes the trafficking of incoming virions into endocytic pathways that are acidified, degradatory and/or otherwise non-productive.

## Perspectives

Since IFITM proteins share a high degree of sequence homology (especially IFITM2 and IFITM3, which differ by only 13 amino acids), future efforts must assess the specific or redundant role for each IFITM family member when describing new activities. Only then can the functional utility of each be properly understood, be it during virus infection or basal cellular homeostasis. Furthermore, it is important to test whether interferon signaling serves as a ‘switch’ to promote antiviral functions over housekeeping ones. We expect that boosting efforts towards understanding the host factors interacting with IFITM proteins will expose novel purposes in cells, which may, in turn, reveal additional ways by which these proteins interfere with virus infections. Cell-based experiments have identified many binding partners of IFITM proteins, but in most cases, it is unclear whether these interactions are direct or specific. Interrogation of potential interactors in transgenic mice will help clarify their precise role and enable prioritization of different hypotheses. In general, increased experimental cross-talk between virology and cell biology will provide much needed opportunities for discovery and will facilitate the development of host-directed therapies targeting IFITM proteins in the setting of infection and cancer.
